# 2-(Biphenyl-4-yl)propan-2-ol

**DOI:** 10.1107/S1600536812003716

**Published:** 2012-02-04

**Authors:** Eric Modau, David C. Liles, Petrus H. van Rooyen

**Affiliations:** a Department of Chemistry, University of Pretoria, Private Bag X20, Hatfield 0028, South Africa

## Abstract

The title compound, C_15_H_16_O, crystallizes with two independent mol­ecules in the asymmetric unit. Due to the space-group symmetry, this results in the formation of a tetra­mer where the four mol­ecules are connected by O—H⋯O hydrogen bonds. The mol­ecules pack parallel to the *c* axis. Both mol­ecules in the asymmetric unit are nonplanar and the dihedral angles between connected aromatic rings in each mol­ecule are 7.96 (12) and 9.75 (13)°. This contrasts with the gas phase density functional theory (DFT) optimized conformation, where this dihedral angle is 39.33°.

## Related literature

For some previous studies of biphenyl derivitives, see: Britton & Gleason (1991 ▶); Britton & Young (2003 ▶); Brock (1980 ▶); Brock & Haller (1980 ▶); Mohamed *et al.* (2003 ▶). For details of *GAUSSIAN03*, see: Frisch *et al.* (2003 ▶).
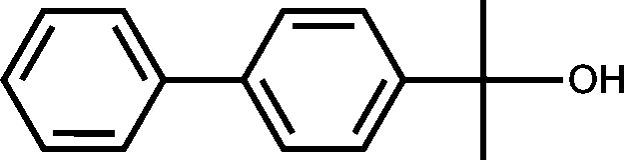


## Experimental

### 

#### Crystal data

C_15_H_16_O
*M* *_r_* = 212.28
Monoclinic, 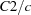

*a* = 12.4406 (14) Å
*b* = 15.5754 (18) Å
*c* = 25.741 (3) Å
β = 102.332 (2)°
*V* = 4872.7 (10) Å^3^
*Z* = 16
Mo *K*α radiation
μ = 0.07 mm^−1^
*T* = 295 K
0.46 × 0.36 × 0.08 mm

#### Data collection

Bruker *P*4 diffractometer with SMART 1000 CCD area detector
Absorption correction: multi-scan (*SADABS*; Bruker, 2001 ▶) *T*_min_ = 0.931, *T*_max_ = 0.994
12927 measured reflections
4590 independent reflections
2859 reflections with *I* > 2σ(*I*)
*R*_int_ = 0.029

#### Refinement

*R*[*F* ^2^ > 2σ(*F* ^2^)] = 0.046
*wR*(*F* ^2^) = 0.147
*S* = 1.01
4590 reflections
391 parameters
3 restraints
All H-atom parameters refined
Δρ_max_ = 0.13 e Å^−3^
Δρ_min_ = −0.14 e Å^−3^

### 

Data collection: *SMART* (Bruker, 2001 ▶); cell refinement: *SAINT* (Bruker, 2001 ▶); data reduction: *SAINT*; program(s) used to solve structure: *SHELXTL* (Sheldrick, 2008 ▶); program(s) used to refine structure: *SHELXTL* and *SHELXL97* (Sheldrick, 2008 ▶); molecular graphics: *ORTEP-3 for Windows* (Farrugia, 1997 ▶), *Mercury* (Macrae *et al.*, 2008 ▶) and *POV-RAY* (Cason, 2004 ▶); software used to prepare material for publication: *SHELXL97* and *PLATON* (Spek, 2009 ▶).

## Supplementary Material

Crystal structure: contains datablock(s) I, global. DOI: 10.1107/S1600536812003716/zq2152sup1.cif

Structure factors: contains datablock(s) I. DOI: 10.1107/S1600536812003716/zq2152Isup2.hkl

Supplementary material file. DOI: 10.1107/S1600536812003716/zq2152Isup3.cml

Additional supplementary materials:  crystallographic information; 3D view; checkCIF report

## Figures and Tables

**Table 1:** Hydrogen-bond geometry (Å, °)

*D*—H⋯*A*	*D*—H	H⋯*A*	*D*⋯*A*	*D*—H⋯*A*
O1—H1*A*⋯O2	0.90 (1)	1.99 (2)	2.804 (2)	150 (4)
O2—H2*A*⋯O1	0.90 (1)	2.09 (4)	2.804 (2)	136 (4)
O1—H1*B*⋯O1^i^	0.87 (3)	1.90 (3)	2.767 (3)	174 (4)
O2—H2*B*⋯O2^i^	0.89 (1)	2.03 (1)	2.926 (3)	177 (4)

Symmetry code: (i) 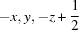.

**Fig. 1.**
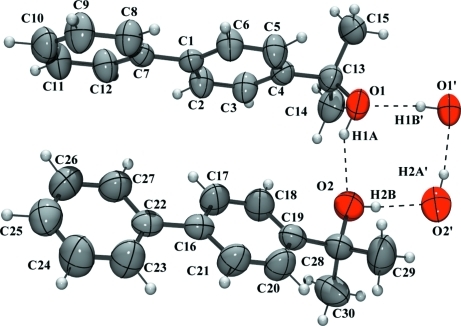
Perspective view of the asymmetric unit of the title compound, with the atom numbering. This shows one of the two possible orientations of the hydrogen bonding scheme. Displacement ellipsoids are shown at the 50% probability level.

**Fig. 2.**
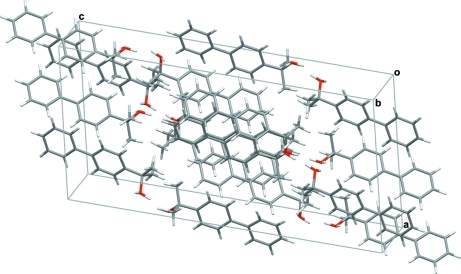
Drawing of the unit cell content of the title compound.

## supplementary crystallographic information

### Comment

The studies of the series of biphenyl derivatives have attracted considerable attention for some time now. This included the *para*-monosubstituted derivatives 4-bromobiphenyl (Brock, 1980) and 4-hydroxylbiphenyl (Brock & Haller, 1980), as well as some *para*-disubstituted derivatives such as 4,4'-dibromobiphenyl (Mohamed *et al.*, 2003), 4,4'-iodocyanobiphenyl (Britton & Gleason, 1991) and 4,4'-dicyanobiphenyl (Britton & Young, 2003). Particular interest has been shown in their packing motifs as well as the inter-ring dihedral angles which are found to be approximately 40° in the solid state in the majority of structures. The structure of the corresponding 2-(4-biphenyl)-2-propanol compound, was undertaken as part of the investigation into the conformational properties of *para* monosubstituted and *para* disubstituted biphenyls. Of significance is that this compound crystallizes in a significantly more planar conformation than what is expected, although it is still non-planar.

2-(4-biphenyl)-2-propanol crystallizes with two independent molecules in the asymmetric unit. The presence of a twofold rotational axis results in the formation of a hydrogen bonded tetramer. The four H atoms of the hydroxyl groups occupy both sets of possible hydrogen positions, illustrated by the two possible bonding schemes (H···O_A_—H···O_B_—H) and (H—O_A_···H—O_B_···H). Both sets of H atom positions were refined with occupancies of 0.5. The two molecules in the asymmetric unit have similar geometrical parameters. The molecules are non-planar: the two aromatic rings in each molecule are slightly twisted around C—C inter ring bond by 7.96 (3)° and 9.75 (3)°. This contrasts to the gas phase DFT (6–31+G**) optimized conformation where this dihedral angle is 39.33° (GAUSSIAN03, Frisch *et al.*, 2003). The anisotropic displacement ellipsoids and atom labelling for the compound is shown in Fig.1. The lengths of the central C—C bonds connecting the two aromatic rings in each of the two molecules are equal to 1.491 (3) and 1.489 (2) Å. The bond length and bond angle are within the expected values. The H···O distances are 1.99 (2), 2.09 (4), 1.90 (3) and 2.034 (11) Å. The molecules pack parallel to the *c* axis (Fig. 2). The volume per non H atom in the crystal is 19.03 Å^3^, in line with that calculated for other biphenyl derivatives structures. This would suggest that the closer packing resulting from the intermolecular hydrogen bonds as well as the more planar biphenyl systems does not significantly change the packing requirements in the crystals.

### Experimental

The title compound was obtained from Aldrich Chemical Co. Inc. Crystals were grown from distilled hexane, acetone, benzene, dichloromethane, chloroform, carbon tetrachloride, and acetonitrile in an attempt to search for multiple polymorphs. Several habits were found, *viz.* prisms, clear plates, and striated plates but all proved to be isostructural. A prism grown from distilled hexane was used for the structure determination.

Geometry optimization for 2-(4-biphenyl)-2-propanol was performed using the program GAUSSIAN03 and applying the B3LYP-functional with the 6-31+G** basis set level (Frisch *et al.*, 2003). This optimized structure displayed no negative vibrational frequencies.

### Refinement

All H atom positions were obtained from difference Fourier maps and were freely refined. Isotropic displacement parameters for the H atoms were set at 1.2 times the equivalent isotropic displacement parameter of the atom to which each H atom is bonded (1.5 times for the methyl H atoms). The two independent molecules, plus two further molecules generated by a crystallographic 2-fold rotation axis, form a hydrogen bonded tetramer. The hydroxyl H atoms involved in the hydrogen bonding are, of necessity, disordered and two H atom positions were observed for each hydroxyl group and each hydrogen position was refined with a sof of 0.5.

### Crystal data

**Table:**

C_15_H_16_O	*F*(000) = 1824
*M**_r_* = 212.28	*D*_x_ = 1.157 Mg m^−^^3^
Monoclinic, *C*2/*c*	Melting point: 366.1 K
Hall symbol: -C 2yc	Mo *K*α radiation, λ = 0.71073 Å
*a* = 12.4406 (14) Å	Cell parameters from 4663 reflections
*b* = 15.5754 (18) Å	θ = 2.4–26.0°
*c* = 25.741 (3) Å	µ = 0.07 mm^−^^1^
β = 102.332 (2)°	*T* = 295 K
*V* = 4872.7 (10) Å^3^	Plate, colourless
*Z* = 16	0.46 × 0.36 × 0.08 mm

### Data collection

**Table:**

Bruker P4 diffractometer with SMART 1000 CCD area detector	4590 independent reflections
Radiation source: fine-focus sealed tube	2859 reflections with *I* > 2σ(*I*)
Graphite monochromator	*R*_int_ = 0.029
Detector resolution: 8.3 pixels mm^-1^	θ_max_ = 26.5°, θ_min_ = 2.4°
φ and ω scans	*h* = −15→7
Absorption correction: multi-scan (*SADABS*; Bruker, 2001)	*k* = −18→15
*T*_min_ = 0.931, *T*_max_ = 0.994	*l* = −31→31
12927 measured reflections	

### Refinement

**Table:**

Refinement on *F*^2^	Primary atom site location: structure-invariant direct methods
Least-squares matrix: full	Secondary atom site location: difference Fourier map
*R*[*F*^2^ > 2σ(*F*^2^)] = 0.046	Hydrogen site location: difference Fourier map
*wR*(*F*^2^) = 0.147	All H-atom parameters refined
*S* = 1.01	*w* = 1/[σ^2^(*F*_o_^2^) + (0.0639*P*)^2^ + 1.7634*P*] where *P* = (*F*_o_^2^ + 2*F*_c_^2^)/3
4590 reflections	(Δ/σ)_max_ = 0.001
391 parameters	Δρ_max_ = 0.13 e Å^−^^3^
3 restraints	Δρ_min_ = −0.14 e Å^−^^3^

### Special details

**Table:**

Geometry. All e.s.d.'s (except the e.s.d. in the dihedral angle between two l.s. planes) are estimated using the full covariance matrix. The cell e.s.d.'s are taken into account individually in the estimation of e.s.d.'s in distances, angles and torsion angles; correlations between e.s.d.'s in cell parameters are only used when they are defined by crystal symmetry. An approximate (isotropic) treatment of cell e.s.d.'s is used for estimating e.s.d.'s involving l.s. planes.
Refinement. Refinement of *F*^2^ against ALL reflections. The weighted *R*-factor *wR* and goodness of fit *S* are based on *F*^2^, conventional *R*-factors *R* are based on *F*, with *F* set to zero for negative *F*^2^. The threshold expression of *F*^2^ > σ(*F*^2^) is used only for calculating *R*-factors(gt) *etc*. and is not relevant to the choice of reflections for refinement. *R*-factors based on *F*^2^ are statistically about twice as large as those based on *F*, and *R*-factors based on ALL data will be even larger.

### Fractional atomic coordinates and isotropic or equivalent isotropic displacement parameters (Å^2^)

**Table:**

	*x*	*y*	*z*	*U*_iso_*/*U*_eq_	Occ. (<1)
C1	0.22391 (12)	0.49260 (10)	0.05345 (6)	0.0485 (4)	
C2	0.28952 (16)	0.44401 (13)	0.09272 (7)	0.0646 (5)	
H2	0.3512 (17)	0.4133 (13)	0.0851 (7)	0.078*	
C3	0.26765 (16)	0.43652 (13)	0.14308 (7)	0.0650 (5)	
H3	0.3152 (16)	0.4000 (13)	0.1686 (8)	0.078*	
C4	0.17907 (13)	0.47641 (11)	0.15649 (7)	0.0530 (4)	
C5	0.11288 (16)	0.52481 (14)	0.11731 (8)	0.0687 (5)	
H5	0.0523 (17)	0.5571 (13)	0.1256 (8)	0.082*	
C6	0.13494 (16)	0.53305 (13)	0.06735 (8)	0.0654 (5)	
H6	0.0899 (16)	0.5687 (13)	0.0427 (8)	0.078*	
C7	0.24761 (13)	0.50013 (10)	−0.00067 (6)	0.0510 (4)	
C8	0.17499 (17)	0.53907 (14)	−0.04217 (8)	0.0715 (6)	
H8	0.1095 (18)	0.5621 (14)	−0.0353 (8)	0.086*	
C9	0.1975 (2)	0.54432 (17)	−0.09240 (9)	0.0839 (7)	
H9	0.1447 (19)	0.5734 (15)	−0.1189 (9)	0.101*	
C10	0.2925 (2)	0.51127 (15)	−0.10229 (8)	0.0790 (6)	
H10	0.3063 (18)	0.5144 (14)	−0.1383 (9)	0.095*	
C11	0.36620 (19)	0.47263 (16)	−0.06195 (8)	0.0795 (6)	
H11	0.4342 (19)	0.4466 (14)	−0.0681 (8)	0.095*	
C12	0.34381 (17)	0.46705 (14)	−0.01181 (8)	0.0693 (5)	
H12	0.3974 (17)	0.4414 (13)	0.0170 (8)	0.083*	
C13	0.15214 (15)	0.46972 (12)	0.21136 (7)	0.0599 (5)	
C14	0.2251 (2)	0.40631 (18)	0.24767 (9)	0.0803 (6)	
H14A	0.222 (2)	0.3454 (19)	0.2307 (10)	0.120*	
H14B	0.199 (2)	0.4014 (17)	0.2819 (11)	0.120*	
H14C	0.301 (2)	0.4270 (17)	0.2564 (10)	0.120*	
C15	0.1579 (2)	0.55713 (16)	0.23792 (10)	0.0855 (7)	
H15A	0.104 (2)	0.6003 (19)	0.2143 (11)	0.128*	
H15B	0.236 (2)	0.5802 (18)	0.2442 (11)	0.128*	
H15C	0.139 (2)	0.5516 (17)	0.2739 (12)	0.128*	
O1	0.03981 (11)	0.44009 (9)	0.20377 (5)	0.0678 (4)	
H1A	0.047 (3)	0.3863 (12)	0.1923 (16)	0.081*	0.50
H1B	0.012 (4)	0.443 (2)	0.2319 (15)	0.081*	0.50
C16	0.00267 (14)	0.24680 (11)	−0.00238 (8)	0.0573 (4)	
C17	0.07693 (17)	0.27485 (15)	0.04215 (9)	0.0787 (6)	
H17	0.1405 (19)	0.3030 (15)	0.0365 (8)	0.094*	
C18	0.05831 (18)	0.26437 (15)	0.09270 (9)	0.0805 (7)	
H18	0.1060 (19)	0.2895 (15)	0.1211 (9)	0.097*	
C19	−0.03521 (14)	0.22565 (11)	0.10169 (8)	0.0608 (5)	
C20	−0.11092 (17)	0.19968 (15)	0.05718 (9)	0.0774 (6)	
H20	−0.1768 (19)	0.1719 (14)	0.0629 (8)	0.093*	
C21	−0.09266 (17)	0.20967 (15)	0.00691 (9)	0.0766 (6)	
H21	−0.1457 (18)	0.1882 (14)	−0.0227 (9)	0.092*	
C22	0.02432 (14)	0.25405 (11)	−0.05698 (8)	0.0594 (5)	
C23	−0.04138 (19)	0.21334 (18)	−0.10009 (10)	0.0887 (7)	
H23	−0.100 (2)	0.1765 (16)	−0.0918 (9)	0.106*	
C24	−0.0217 (2)	0.2200 (2)	−0.15056 (11)	0.1001 (8)	
H24	−0.064 (2)	0.1888 (18)	−0.1788 (11)	0.120*	
C25	0.0651 (2)	0.26587 (16)	−0.15981 (11)	0.0867 (7)	
H25	0.0803 (19)	0.2704 (15)	−0.1976 (10)	0.104*	
C26	0.1323 (2)	0.30524 (17)	−0.11834 (11)	0.0952 (7)	
H26	0.195 (2)	0.3407 (17)	−0.1234 (10)	0.114*	
C27	0.1118 (2)	0.29994 (15)	−0.06773 (10)	0.0849 (7)	
H27	0.1563 (19)	0.3281 (16)	−0.0394 (10)	0.102*	
C28	−0.05169 (16)	0.20597 (13)	0.15723 (8)	0.0696 (5)	
C29	−0.1704 (2)	0.2169 (2)	0.16242 (13)	0.1090 (10)	
H29A	−0.214 (3)	0.168 (2)	0.1416 (13)	0.163*	
H29B	−0.173 (3)	0.209 (2)	0.1994 (14)	0.163*	
H29C	−0.191 (3)	0.276 (2)	0.1486 (14)	0.163*	
C30	−0.0115 (3)	0.11470 (18)	0.17181 (12)	0.1097 (10)	
H30A	−0.053 (3)	0.075 (2)	0.1448 (14)	0.165*	
H30B	−0.023 (3)	0.103 (2)	0.2069 (14)	0.165*	
H30C	0.070 (3)	0.116 (2)	0.1726 (13)	0.165*	
O2	0.01619 (14)	0.26127 (10)	0.19536 (6)	0.0871 (5)	
H2A	0.000 (4)	0.3133 (16)	0.1811 (19)	0.104*	0.50
H2B	0.007 (5)	0.259 (3)	0.2287 (9)	0.104*	0.50

### Atomic displacement parameters (Å^2^)

**Table:**

	*U*^11^	*U*^22^	*U*^33^	*U*^12^	*U*^13^	*U*^23^
C1	0.0478 (9)	0.0447 (9)	0.0519 (10)	−0.0059 (7)	0.0082 (7)	−0.0009 (7)
C2	0.0578 (11)	0.0760 (13)	0.0614 (11)	0.0171 (9)	0.0157 (9)	0.0078 (10)
C3	0.0634 (11)	0.0742 (13)	0.0569 (11)	0.0143 (10)	0.0115 (9)	0.0145 (10)
C4	0.0543 (10)	0.0526 (10)	0.0523 (9)	−0.0055 (8)	0.0116 (8)	0.0004 (8)
C5	0.0636 (11)	0.0817 (14)	0.0641 (12)	0.0198 (10)	0.0210 (10)	0.0090 (10)
C6	0.0638 (11)	0.0740 (13)	0.0585 (11)	0.0184 (9)	0.0134 (9)	0.0121 (9)
C7	0.0531 (9)	0.0477 (9)	0.0508 (9)	−0.0077 (7)	0.0080 (7)	−0.0051 (8)
C8	0.0641 (12)	0.0897 (15)	0.0605 (12)	0.0096 (11)	0.0129 (10)	0.0086 (10)
C9	0.0842 (15)	0.1084 (19)	0.0564 (12)	0.0126 (13)	0.0089 (11)	0.0125 (12)
C10	0.0921 (16)	0.0942 (16)	0.0522 (12)	−0.0060 (12)	0.0189 (11)	−0.0031 (11)
C11	0.0799 (14)	0.0985 (17)	0.0646 (13)	0.0091 (12)	0.0253 (11)	−0.0048 (12)
C12	0.0671 (12)	0.0843 (14)	0.0565 (11)	0.0109 (10)	0.0133 (9)	0.0008 (10)
C13	0.0632 (11)	0.0641 (11)	0.0536 (10)	−0.0071 (9)	0.0155 (8)	0.0003 (8)
C14	0.0842 (15)	0.1001 (18)	0.0554 (12)	0.0009 (13)	0.0121 (11)	0.0148 (12)
C15	0.1139 (19)	0.0788 (15)	0.0682 (14)	−0.0162 (13)	0.0292 (14)	−0.0145 (12)
O1	0.0656 (8)	0.0812 (9)	0.0623 (8)	−0.0060 (7)	0.0260 (6)	−0.0004 (7)
C16	0.0517 (10)	0.0466 (10)	0.0703 (12)	−0.0018 (8)	0.0058 (8)	−0.0028 (8)
C17	0.0620 (12)	0.0926 (16)	0.0821 (15)	−0.0288 (11)	0.0170 (11)	−0.0167 (12)
C18	0.0665 (13)	0.0997 (17)	0.0731 (14)	−0.0284 (11)	0.0101 (11)	−0.0239 (12)
C19	0.0587 (10)	0.0523 (10)	0.0711 (12)	−0.0050 (8)	0.0129 (9)	−0.0118 (9)
C20	0.0628 (12)	0.0896 (15)	0.0781 (15)	−0.0270 (11)	0.0113 (11)	−0.0060 (12)
C21	0.0636 (12)	0.0893 (15)	0.0710 (14)	−0.0240 (11)	0.0012 (10)	−0.0047 (11)
C22	0.0541 (10)	0.0458 (10)	0.0749 (13)	0.0044 (8)	0.0063 (9)	0.0015 (9)
C23	0.0703 (14)	0.1149 (19)	0.0760 (15)	−0.0245 (13)	0.0046 (11)	−0.0042 (13)
C24	0.0944 (18)	0.129 (2)	0.0716 (16)	−0.0171 (16)	0.0056 (13)	−0.0087 (15)
C25	0.0967 (17)	0.0844 (16)	0.0806 (16)	0.0126 (13)	0.0224 (14)	0.0113 (13)
C26	0.1011 (18)	0.0903 (17)	0.102 (2)	−0.0217 (14)	0.0382 (16)	0.0034 (15)
C27	0.0867 (15)	0.0809 (15)	0.0872 (17)	−0.0247 (12)	0.0190 (13)	−0.0080 (12)
C28	0.0766 (12)	0.0622 (12)	0.0710 (13)	−0.0150 (10)	0.0182 (10)	−0.0169 (10)
C29	0.0908 (18)	0.147 (3)	0.098 (2)	−0.0350 (18)	0.0402 (16)	−0.0268 (19)
C30	0.182 (3)	0.0733 (17)	0.0754 (16)	−0.0030 (19)	0.0314 (19)	−0.0039 (13)
O2	0.0979 (11)	0.0883 (11)	0.0762 (11)	−0.0292 (9)	0.0213 (9)	−0.0268 (9)

### Geometric parameters (Å, º)

**Table:**

C1—C2	1.381 (2)	C16—C17	1.380 (3)
C1—C6	1.385 (2)	C16—C21	1.385 (3)
C1—C7	1.489 (2)	C16—C22	1.491 (3)
C2—C3	1.385 (3)	C17—C18	1.379 (3)
C2—H2	0.96 (2)	C17—H17	0.94 (2)
C3—C4	1.372 (2)	C18—C19	1.373 (3)
C3—H3	0.97 (2)	C18—H18	0.92 (2)
C4—C5	1.382 (2)	C19—C20	1.379 (3)
C4—C13	1.523 (2)	C19—C28	1.518 (3)
C5—C6	1.377 (3)	C20—C21	1.370 (3)
C5—H5	0.97 (2)	C20—H20	0.97 (2)
C6—H6	0.93 (2)	C21—H21	0.96 (2)
C7—C8	1.383 (2)	C22—C27	1.379 (3)
C7—C12	1.388 (3)	C22—C23	1.384 (3)
C8—C9	1.383 (3)	C23—C24	1.376 (3)
C8—H8	0.94 (2)	C23—H23	0.98 (2)
C9—C10	1.362 (3)	C24—C25	1.357 (4)
C9—H9	0.95 (2)	C24—H24	0.94 (3)
C10—C11	1.369 (3)	C25—C26	1.354 (4)
C10—H10	0.98 (2)	C25—H25	1.03 (2)
C11—C12	1.380 (3)	C26—C27	1.382 (3)
C11—H11	0.98 (2)	C26—H26	0.99 (3)
C12—H12	0.97 (2)	C27—H27	0.93 (2)
C13—O1	1.445 (2)	C28—O2	1.437 (2)
C13—C15	1.518 (3)	C28—C29	1.521 (3)
C13—C14	1.520 (3)	C28—C30	1.527 (3)
C14—H14A	1.04 (3)	C29—H29A	1.01 (4)
C14—H14B	1.00 (3)	C29—H29B	0.97 (4)
C14—H14C	0.97 (3)	C29—H29C	1.00 (3)
C15—H15A	1.04 (3)	C30—H30A	0.98 (4)
C15—H15B	1.01 (3)	C30—H30B	0.96 (3)
C15—H15C	1.01 (3)	C30—H30C	1.01 (4)
O1—H1A	0.900 (10)	O2—H2A	0.895 (10)
O1—H1B	0.87 (3)	O2—H2B	0.893 (10)
			
C2—C1—C6	116.10 (16)	C17—C16—C21	115.77 (19)
C2—C1—C7	121.57 (15)	C17—C16—C22	122.35 (17)
C6—C1—C7	122.32 (15)	C21—C16—C22	121.87 (17)
C1—C2—C3	121.83 (17)	C18—C17—C16	121.90 (19)
C1—C2—H2	119.8 (12)	C18—C17—H17	121.1 (13)
C3—C2—H2	118.4 (12)	C16—C17—H17	117.0 (14)
C4—C3—C2	121.75 (17)	C19—C18—C17	122.01 (19)
C4—C3—H3	120.2 (12)	C19—C18—H18	118.4 (14)
C2—C3—H3	118.0 (12)	C17—C18—H18	119.2 (14)
C3—C4—C5	116.69 (16)	C18—C19—C20	116.20 (19)
C3—C4—C13	123.36 (16)	C18—C19—C28	122.48 (17)
C5—C4—C13	119.96 (16)	C20—C19—C28	121.16 (17)
C6—C5—C4	121.73 (17)	C21—C20—C19	122.00 (19)
C6—C5—H5	118.5 (12)	C21—C20—H20	120.8 (13)
C4—C5—H5	119.7 (12)	C19—C20—H20	117.2 (13)
C5—C6—C1	121.90 (17)	C20—C21—C16	122.09 (19)
C5—C6—H6	118.7 (12)	C20—C21—H21	119.2 (13)
C1—C6—H6	119.4 (12)	C16—C21—H21	118.6 (13)
C8—C7—C12	116.94 (17)	C27—C22—C23	115.7 (2)
C8—C7—C1	122.00 (16)	C27—C22—C16	122.54 (18)
C12—C7—C1	121.06 (16)	C23—C22—C16	121.79 (18)
C9—C8—C7	121.3 (2)	C24—C23—C22	121.9 (2)
C9—C8—H8	120.7 (13)	C24—C23—H23	122.5 (14)
C7—C8—H8	118.1 (13)	C22—C23—H23	115.4 (14)
C10—C9—C8	120.6 (2)	C25—C24—C23	120.9 (2)
C10—C9—H9	122.5 (14)	C25—C24—H24	118.5 (17)
C8—C9—H9	116.8 (14)	C23—C24—H24	120.4 (17)
C9—C10—C11	119.5 (2)	C26—C25—C24	118.7 (3)
C9—C10—H10	119.6 (13)	C26—C25—H25	120.4 (13)
C11—C10—H10	121.0 (13)	C24—C25—H25	120.9 (13)
C10—C11—C12	120.0 (2)	C25—C26—C27	120.6 (2)
C10—C11—H11	121.4 (13)	C25—C26—H26	121.5 (15)
C12—C11—H11	118.6 (13)	C27—C26—H26	117.8 (15)
C11—C12—C7	121.67 (19)	C22—C27—C26	122.1 (2)
C11—C12—H12	119.8 (12)	C22—C27—H27	116.9 (15)
C7—C12—H12	118.5 (12)	C26—C27—H27	121.0 (15)
O1—C13—C15	107.35 (17)	O2—C28—C19	110.14 (15)
O1—C13—C14	108.02 (16)	O2—C28—C29	108.26 (18)
C15—C13—C14	109.97 (18)	C19—C28—C29	112.9 (2)
O1—C13—C4	107.11 (14)	O2—C28—C30	106.0 (2)
C15—C13—C4	110.96 (16)	C19—C28—C30	108.45 (18)
C14—C13—C4	113.18 (16)	C29—C28—C30	110.8 (2)
C13—C14—H14A	111.6 (14)	C28—C29—H29A	106.7 (19)
C13—C14—H14B	108.9 (15)	C28—C29—H29B	108 (2)
H14A—C14—H14B	108 (2)	H29A—C29—H29B	107 (3)
C13—C14—H14C	110.4 (16)	C28—C29—H29C	104 (2)
H14A—C14—H14C	111 (2)	H29A—C29—H29C	116 (3)
H14B—C14—H14C	107 (2)	H29B—C29—H29C	114 (3)
C13—C15—H15A	110.6 (15)	C28—C30—H30A	108 (2)
C13—C15—H15B	109.9 (17)	C28—C30—H30B	107 (2)
H15A—C15—H15B	109 (2)	H30A—C30—H30B	113 (3)
C13—C15—H15C	109.8 (16)	C28—C30—H30C	105 (2)
H15A—C15—H15C	110 (2)	H30A—C30—H30C	114 (3)
H15B—C15—H15C	107 (2)	H30B—C30—H30C	109 (3)
C13—O1—H1A	100 (3)	C28—O2—H2A	102 (3)
C13—O1—H1B	114 (3)	C28—O2—H2B	117 (3)
H1A—O1—H1B	114 (4)	H2A—O2—H2B	111 (5)
			
C6—C1—C2—C3	−0.2 (3)	C21—C16—C17—C18	1.6 (3)
C7—C1—C2—C3	−179.76 (17)	C22—C16—C17—C18	−177.0 (2)
C1—C2—C3—C4	0.6 (3)	C16—C17—C18—C19	−0.2 (4)
C2—C3—C4—C5	−0.3 (3)	C17—C18—C19—C20	−1.6 (3)
C2—C3—C4—C13	179.94 (18)	C17—C18—C19—C28	173.9 (2)
C3—C4—C5—C6	−0.3 (3)	C18—C19—C20—C21	1.8 (3)
C13—C4—C5—C6	179.45 (18)	C28—C19—C20—C21	−173.8 (2)
C4—C5—C6—C1	0.7 (3)	C19—C20—C21—C16	−0.3 (4)
C2—C1—C6—C5	−0.4 (3)	C17—C16—C21—C20	−1.4 (3)
C7—C1—C6—C5	179.15 (18)	C22—C16—C21—C20	177.3 (2)
C2—C1—C7—C8	171.37 (18)	C17—C16—C22—C27	−9.5 (3)
C6—C1—C7—C8	−8.1 (3)	C21—C16—C22—C27	171.9 (2)
C2—C1—C7—C12	−7.8 (3)	C17—C16—C22—C23	169.1 (2)
C6—C1—C7—C12	172.69 (18)	C21—C16—C22—C23	−9.5 (3)
C12—C7—C8—C9	0.2 (3)	C27—C22—C23—C24	−1.4 (4)
C1—C7—C8—C9	−178.97 (19)	C16—C22—C23—C24	179.9 (2)
C7—C8—C9—C10	−0.2 (4)	C22—C23—C24—C25	1.3 (4)
C8—C9—C10—C11	−0.1 (4)	C23—C24—C25—C26	0.0 (4)
C9—C10—C11—C12	0.3 (4)	C24—C25—C26—C27	−1.0 (4)
C10—C11—C12—C7	−0.2 (3)	C23—C22—C27—C26	0.3 (3)
C8—C7—C12—C11	0.0 (3)	C16—C22—C27—C26	179.0 (2)
C1—C7—C12—C11	179.18 (18)	C25—C26—C27—C22	0.9 (4)
C3—C4—C13—O1	−125.65 (18)	C18—C19—C28—O2	23.1 (3)
C5—C4—C13—O1	54.6 (2)	C20—C19—C28—O2	−161.64 (19)
C3—C4—C13—C15	117.5 (2)	C18—C19—C28—C29	144.2 (2)
C5—C4—C13—C15	−62.3 (2)	C20—C19—C28—C29	−40.5 (3)
C3—C4—C13—C14	−6.7 (3)	C18—C19—C28—C30	−92.5 (3)
C5—C4—C13—C14	173.51 (19)	C20—C19—C28—C30	82.7 (3)

### Hydrogen-bond geometry (Å, º)

**Table:**

*D*—H···*A*	*D*—H	H···*A*	*D*···*A*	*D*—H···*A*
O1—H1*A*···O2	0.90 (1)	1.99 (2)	2.804 (2)	150 (4)
O2—H2*A*···O1	0.90 (1)	2.09 (4)	2.804 (2)	136 (4)
O1—H1*B*···O1^i^	0.87 (3)	1.90 (3)	2.767 (3)	174 (4)
O2—H2*B*···O2^i^	0.89 (1)	2.03 (1)	2.926 (3)	177 (4)

Symmetry code: (i) −*x*, *y*, −*z*+1/2.
